# Dataset on thermal comfort, perceived stress, and anxiety in university students under confinement due to COVID-19 in a hot and humid region of Mexico

**DOI:** 10.1016/j.dib.2022.107996

**Published:** 2022-02-25

**Authors:** C. Ramírez-Dolores, L.A. Lugo-Ramírez, B.A. Hernández-Cortaza, G. Alcalá, J. Lara-Valdés, J. Andaverde

**Affiliations:** a Institute for Renewable Energy, National Autonomous University of Mexico, Morelos, Mexico; b Center for the Human and Integral Development of University Students, University of Veracruz, Coatzacoalcos, Mexico; c Faculty of Nursing, University of Veracruz, Veracruz, Mexico; d Center for Research in Energy and Sustainable Resources, University of Veracruz, Veracruz, Mexico; e Faculty of Medicine, University of Veracruz, Veracruz, Mexico; f Faculty of Chemical Sciences, University of Veracruz, Veracruz, Mexico

**Keywords:** Thermal sensation, Humidity sensation, Stress perceived, Anxiety, COVID-19

## Abstract

This dataset was compiled to estimate the levels of thermal comfort and mental health in a sample group of university students confined due to the COVID-19 pandemic. By the time research was carried out, these students of a hot and humid region of Mexico, had already spent 200 days on distance learning using online platforms. A total of 324 records were documented with a final sample of 316 valid participants. The total records were collected directly from the students through a web platform (Microsoft forms). This data set can be used to generate correlations between mental health, thermal comfort, and individual characteristics in the study population that will allow to identify the influence of the built environment and local climate on the levels of stress and anxiety that university students experienced under confinement. It can also be used to issue recommendations to improve the quality of built spaces and for the construction of adaptive models of thermal comfort considering mental health as a study variable*.*

## Specifications Table

**Table:** 

Subject	Engineering, psychology.
Specific subject area	Thermal comfort, mental health, university students.
Type of data	Text, Table, Graph, Excel file.
How the data were acquired	A survey was used to collect data, which included a questionnaire of personal information (to obtain the sociodemographic information, school data, Internet connection, and health characteristics); Adaptation of the Questionnaire of Indoor Thermal Environments; The Scale of Stress Perceived of Cohen PSS-14; and Beck Anxiety Inventory (BAI). This survey was applied online on the platform Microsoft Forms and subsequently exported into its Excel file (.xls) version for processing.
Data format	Raw and analyzed.
Parameters for data collection	The study population consisted of students from a public university. The data was collected during the first month of the academic period February – July 2021. The students were using the online learning platforms throughout this semester due to the ongoing coronavirus pandemic (COVID-19).
Description of data collection	A web-based questionnaire on thermal comfort and mental health was developed on the online survey platform Microsoft Forms and promoted through the social networks of a university community. In addition, the online questionnaire was given to different professors who were asked to distribute the questionnaire's link to their students.
Data source location	A public University (protecting the confidentiality of the institution) City: Coatzacoalcos, Veracruz Country: Mexico
Data accessibility	Dataset has been uploaded on Mendeley Repository name: Mendeley repository Direct URL to data: https://data.mendeley.com/datasets/hp7knm4zp9/3 DOI: 10.17632/hp7knm4zp9.2
Related research article	N/A.

## Value of the Data

• The dataset will be useful to analyze the correlations between thermal comfort and mental health of university students facing COVID-19 confinement.
• The dataset can be used to identify the different comfort and discomfort profiles through thermal sensation assessment and health information.
• The dataset will be useful for researchers willing to compare the results against similar data from other regions of the world.
• The dataset will be useful to establish a precedent for COVID-19-derived effects on the thermal responses of individuals.
• The dataset will be useful for researchers who want to describe aspects of health and mental health in university students under confinement.
• The dataset contributes to predict the influence of environmental aspects on mental health in a confinement setting.

## 1 Data Description

The World Health Organization declared the COVID-19 outbreak a public health emergency of international concern on January 30, 2020, since it has spread worldwide, affecting a large number of people [1]. According to the United Nations Educational and Cultural Organization (UNESCO), one year after the onset of the COVID-19 pandemic, almost half of the world's learners continued to be affected by the partial or total closure of schools [2]. Regarding health effects, studies such as that of Ozamiz et al [3]. mention high levels of stress and anxiety in the first phase of the COVID-19 outbreak; severe and extremely severe levels of stress and anxiety were reported in the population, with the 18-to-25 age group having the highest prevalence of extremely severe levels. Likewise, considering confinement as the obligation to remain in their homes, environmental conditions become a factor that should be considered as a mediator in the behavior and health of the individual.

This dataset contains data for the estimation of thermal comfort levels (thermal and humidity) and mental health (stress perceived and anxiety) in a group of university students in the South of Mexico, with ages between 18 and 43 years old and an average of 20. The data was collected through a web platform (Microsoft Forms) where anonymous users answered each question. Then, the information was processed obtaining 316 records (172 females and 144 males) and 2 independent sets: the first set refers to thermal comfort and the second set to mental health, respectively. The surveys were generated and analyzed based on the scales suggested by the original authors of the instruments. The raw data is in Mendeley dataset which can be accessed through the following link: https://data.mendeley.com/datasets/hp7knm4zp9/3

The data about the sociodemographic status, school, internet connection, and health aspects were aimed to characterize the study population. It contained: gender, age, marital status, number of cohabitants, perception of the relationship with cohabitants (Table 1); internet access, type of internet connection, quality of internet connection (Table 2), diagnosed physical and/or mental illnesses (Table 3); consumption of psychoactive substances (alcohol, tobacco, cannabis, cocaine, non-prescription drugs, coffee, energy drinks) (Table 4); aspects related to health lifestyles (hours of sleep, number of meals per day, number of hours dedicated to physical activity per week) (Table 5); aspects related to environmental conditions and thermal comfort (Table 6). All these indicators were considered in the context of COVID-19 confinement.

**Table 1:** Demographic information.

Data	Relative frequency (f)	Absolute frequency (%)
Gender		
Male	144	45.6
Females	172	54.4
Age		
18–23	289	91.5
24–28	20	6.3
29–33	5	1.6
34–38	1	0.3
39–43	1	0.3
Marital status		
Unmarried	311	98.4
Married	3	0.9
Divorced	1	0.3
Free union	1	0.3
Number of partners		
0–3	189	59.8
4–6	122	38.6
7–9	3	0.9
10–12	1	0.3
13–15	1	0.3
Perception of the relationship with partners		
Good	236	74.7
Regular	80	25.3
Poor	0	0

**Table 2:** Data referring to the internet connection in the face of confinement for COVID-19.

Data	Relative frequency (f)	Absolute frequency (%)
Internet Access		
No	16	5.1
Yes	300	94.9
Connection type		
Mobile data	21	6.6
Home wireless network (Wi-Fi)	283	89.6
Public place wireless (Wi-Fi) network	3	0.9
Wired network at home	9	2.8
Internet connection quality		
Good	73	23.1
Regular	193	61.1
Poor	50	15.8

**Table 3:** Medically diagnosed physical and mental illnesses.

	Presence		Absence	
Diseases	f	%	f	%
*Physical*				
Mellitus diabetes	1	0.3	315	99.7
Arterial hypertension	4	1.3	312	98.7
Sexually transmitted infections	1	0.3	315	99.7
Disability	16	5.1	300	94.9
Other physical illness	27	8.5	289	91.5
*Mental*				
Schizophrenia	1	0.3	315	99.7
Major depression syndrome	7	2.2	309	97.8
Generalized anxiety disorder	32	10.1	284	89.9
Another mental illness	11	3.5	305	96.5

*Note:* f - relative frequency; % - absolute frequency.

**Table 4:** Data regarding the consumption of psychoactive substances before confinement for COVID-19.

	Consumption		Do not consume	
Psychoactive substances	f	%	f	%
Alcohol	90	28.5	226	71.5
Tobacco	34	10.8	282	89.2
Cannabis	12	3.8	304	96.2
Cocaine	1	0.3	315	99.7
Non-prescription drugs	22	7.0	294	93.0
Coffee	233	73.7	83	26.3
Energy drinks	83	26.3	233	73.7

*Note:* f - relative frequency; % - absolute frequency.

**Table 5:** Descriptive statistics regarding health lifestyles in confinement for COVID-19.

Data	Min	Max	Mean	Median	SD	IR
Number of usual hours of sleep per day	1	12	6.32	6.00	1.48	2
Number of usual meals per day	1	10	2.72	3.00	1.08	9
Number of hours dedicated to physical activity in the week	0	45	3.11	2.00	4.74	5

*Note:* SD - standard deviation; IR - interquartile range.

**Table 6:** Data about environmental conditions regarding thermal comfort.

Data	Relative frequency (f)	Absolute frequency (%)
*Data of residence*		
*Length of residence (year(s))*		
0–5	67	21.2
6–10	36	11.4
11–15	47	14.9
16–20	106	33.5
21–25	53	16.8
26–30	4	1.3
31–35	2	0.6
> 35	1	0.3
*Data of dressing*		
Present dressing: Upper:		
Shirt	88	27.8
T-shirt	104	32.9
Light blouse	110	34.8
Suit and tie	1	0.3
Coat, sweater, sweatshirt	3	0.9
Dress	3	2.2
None	7	0.9
Present dressing: Lower:		
Jeans	80	25.3
Shorts	219	69.3
Dress	5	1.6
Skirt	6	1.9
Other	6	1.9
Present dressing: Socks:		
Socks (light / short)	81	25.6
Socks (long / thick)	14	4.4
None	221	69.9
*Data of room*		
Time spent in this room		
Morning	61	19.3
Noon	20	6.3
Afternoon	182	57.6
Evening	53	16.8
Habit for window opening		
Frequently	215	68.0
Occasionally	60	19.0
Seldom	29	9.2
Do not have windows	12	3.8
Overall acceptance of thermal environments		
Absolutely unacceptable	14	4.4
Unacceptably	20	6.4
Slightly unacceptable	63	19.9
Slightly acceptable	96	30.4
Acceptable	115	36.4
Absolutely acceptable	8	2.5

Moreover, in the case of the main variables of thermal comfort, the Thermal sensation scale and Humidity sensation scale of the Adaptation of the Questionnaire of Indoor Thermal Environments [4] were applied; for mental health, Cohen's Scale of Stress Perceived PSS-14 [5]; and the Beck Anxiety Inventory (BAI) [6] were applied. These are described below in Table 7.

**Table 7:** Description of the scales (measuring instruments).

Thermal sensation scale		Humidity sensation scale		The Scale of Stress Perceived of Cohen PSS-14	Beck Anxiety Inventory
+3	Hot	+3	Very humid	It consists of 14 items. Seven are worded negative (1, 2, 3, 8, 11, 12, and 14), and the remaining seven are positive (4, 5, 6, 7, 9, 10, and 13). Each item was rated on a five-point Likert-type scale (0 = never to 4 = very often). Total scores were calculated after reversing positive items' scores and then summing up all scores. Possible total scores range from 0 to 56. A higher score indicates greater stress.	It consists of 21 items that evaluate symptoms of anxiety on a four-point Likert scale ranging from 0 = “not at all” to 3 = “severely.” The anxiety level was scored using ordinal categories: normal (0–7 points), mild (8–15), moderate (16–25) and severe (26–63).
+2	Warm	+2	Humid		
+1	Slightly warm	+1	Slightly humid		
0	Neutral	0	Neutral		
1	Slightly cool	1	Slightly dry		
2	Cool	2	Dry		
3	Cold	3	Very dry		

Table 8 presents the general descriptive data corresponding to each set of the main variables. These data are numerical and continuous, so they can be used for correlation, prediction, segmentation, and association analyses. The data is available in Microsoft Excel file format.

**Table 8:** General descriptions of thermal comfort and mental health*.*

	N	Min	Max	Median	Mean	SD	IR
*Perceived stress*	316	3	4	27.00	26.28	7.90	11
*Anxiety*	316	0	53	11.00	14.37	11.54	15
*Thermal sensation*	316	-2	3	2.00	1.46	1.40	3
*Humidity sensation*	316	-3	3	0.00	0.33	1.26	1

*Note:* SD - standard deviation; IR - interquartile range.

## 2 Experimental Design, Materials and Methods

This study was descriptive and cross-sectional. The data was collected through a survey applied to students from a public university who were using online learning platforms during the pandemic (COVID-19). They were in a hot and humid region of Mexico.

### 2.1 Materials

A survey was used for data collection. It was comprised of:

• A questionnaire expressly developed for this study inquiring about personal information such as sociodemographic, school status, Internet connection, and health characteristics. Developed for the purpose of this study.
• Adaptation of the Questionnaire of Indoor Thermal Environments: questions about the environment where they were located, their clothing, perception of the environment, and perception of thermal sensation and humidity [4].
• The Scale of Stress Perceived of Cohen [PSS-14], that measures the degree to which life situations are perceived as stressful. This consists of seven items worded negatively: 1, 2, 3, 8, 11, 12, and 14, and the remaining seven worded positively: 4, 5, 6, 7, 9, 10, and 13. Each item was rated on a five-point Likert-type scale 0 = never to 4 = very often. Total scores were calculated after reversing positive items' scores and then summing up all scores. Possible total scores for PSS-14 range from 0 to 56. A higher score indicates greater stress [7].
• Beck Anxiety Inventory (BAI). It consists of 21 items that evaluate symptoms of anxiety on a four-point Likert scale ranging from 0 = “not at all” to 3 = “severely.” The anxiety level was scored using ordinal categories: normal (0–7 points), mild (8–15), moderate (16–25) and severe (26–63) [8,9].

## Ethics Statements

This study complied with the General Health Law on Research. All procedures performed in this work followed the ethical standards of the institutional committee and the Helsinki declaration and was approved by the ethics committee of the Faculty of Chemical Sciences of the University of Veracruz's Coatzacoalcos Campus, with registration number UV-CA-540-RESEARCH006. Informed consent was obtained from all participants and participant data has been fully anonymized and confidential.

## CRediT authorship contribution statement

**C. Ramírez-Dolores:** Writing – original draft, Formal analysis, Validation. **L.A. Lugo-Ramírez:** Data curation, Writing – review & editing. **B.A. Hernández-Cortaza:** Conceptualization, Methodology. **G. Alcalá:** Visualization, Resources. **J. Lara-Valdés:** Software, Investigation. **J. Andaverde:** Supervision, Funding acquisition, Project administration.

## Declaration of Competing Interest

The authors declare that they have no known competing financial interests or personal relationships that could have appeared to influence the work reported in this paper.
